# Influence of Anticoagulants and Heparin Contaminants on the Suitability of MMP-9 as a Blood-Derived Biomarker

**DOI:** 10.3390/ijms251810106

**Published:** 2024-09-20

**Authors:** Daniela Küper, Josefin Klos, Friederike Kühl, Rozan Attili, Korbinian Brand, Karin Weissenborn, Ralf Lichtinghagen, René Huber

**Affiliations:** 1 Institute of Clinical Chemistry and Laboratory Medicine, Hannover Medical School, 30625 Hannover, Germany; d.kueper@marien-apotheke-hannover.de (D.K.); josefin.klos@stud.mh-hannover.de (J.K.); kuehl.friederike@mh-hannover.de (F.K.); rozana@hebron.edu (R.A.); brand.korbinian@mh-hannover.de (K.B.); lichtinghagen.ralf@mh-hannover.de (R.L.); 2 Faculty of Pharmacy and Medical Sciences, Hebron University, Hebron 711, Palestine; 3 Department of Neurology, Hannover Medical School, 30625 Hannover, Germany; weissenborn.karin@mh-hannover.de

**Keywords:** blood sampling, MMP-9, anticoagulants, heparin, contaminants, over-sulfated chondroitin sulfate

## Abstract

In contrast to other common anticoagulants such as citrate and low-molecular-weight heparin (LMWH), high-molecular-weight heparin (HMWH) induces the expression of matrix metalloproteinase (MMP)-9, which is also measured as a biomarker for stroke in blood samples. Mechanistically, HMWH-stimulated T cells produce cytokines that induce monocytic MMP-9 expression. Here, the influence of further anticoagulants (Fondaparinux, Hirudin, and Alteplase) and the heparin-contaminating glycosaminoglycans (GAG) hyaluronic acid (HA), dermatan sulfate (DS), chondroitin sulfate (CS), and over-sulfated CS (OSCS) on MMP-9 was analyzed to assess its suitability as a biomarker under various conditions. Therefore, starved Jurkat T cells were stimulated with anticoagulants/contaminants. Subsequently, starved monocytic THP-1 cells were incubated with the conditioned Jurkat supernatant, and MMP-9 mRNA levels were monitored (quantitative (q)PCR). Jurkat-derived mediators secreted in response to anticoagulants/contaminants were also assessed (proteome profiler array). The supernatants of HMWH-, Hirudin-, CS-, and OSCS-treated Jurkat cells comprised combinations of activating mediators and led to a significant (in the case of OSCS, dramatic) MMP-9 induction in THP-1. HA induced MMP-9 only in high concentrations, while LMWH, Fondaparinux, Alteplase, and DS had no effect. This indicates that depending on molecular weight and charge (but independent of anticoagulant activity), anticoagulants/contaminants provoke the expression of T-cell-derived cytokines/chemokines that induce monocytic MMP-9 expression, thus potentially impairing the diagnostic validity of MMP-9.

## 1. Introduction

Unfractionated, i.e., high-molecular-weight heparin (HMWH) is a long, linear, and highly sulfated glycosaminoglycan (GAG), with an average molecular weight (MW) of 16 kDa (range: 2–40 kDa). HMWH is exclusively generated by mast cells and predominantly obtained from porcine (more rarely, bovine) intestinal mucosa [1,2]. Though representing a remarkably heterogeneous natural product clinically used for the first time in the 1930s, heparin is still the most prevalent anticoagulant [3] due to low costs, effective speed, reversibility, and—most importantly—its remarkable anticoagulant efficacy [4,5]. The latter is based on its interaction with antithrombin III (ATIII), thus predominantly inhibiting factors FXa and FIIa (i.e., thrombin) [3]. HMWH is especially used for blood sampling [6] but also systemically applied for different therapeutic purposes [7], e.g., prophylaxis and/or the treatment of sepsis [8], acute coronary syndromes, or renal failure, due to its advantages in terms of reversibility and hepatic clearance [3]. Because of its animal origin and non-standardized preparation, HMWH is prone to adulteration and contamination, with severe potential consequences, either for the validity of laboratory parameters assessed in lithium heparin tubes or—if systemically applied—for the life and health of HMWH-treated patients [3,9].

According to the current versions of the European Pharmacopoeia [10] and the United States Pharmacopeia [11], contamination with strictly limited amounts of preparation-related natural by-products, such as dermatan sulfate (DS) and chondroitin sulfate (CS), is regarded as acceptable (up to 2% in total), while higher levels or contamination/adulteration with other (especially chemically modified) substances are not allowed. The long unrecognized presence of semi-synthetically produced over-sulfated chondroitin sulfate (OSCS) in the HMWH batches of different manufacturers, for instance, led to several deaths and severe adverse effects including arrhythmia, hypotension, and anaphylactic reactions in 2007–2008 [2,12]. However, other contaminants and impurities such as hyaluronic acid (HA) [9], histamine [13], ethylenediaminetetraacetic acid (EDTA) [14], heparan sulfate [9,15], as well as water, ethanol, and other residual solvents [16] have also been described in varying amounts (either alone or in combination).

The impact of anticoagulants and their quality on the diagnostic validity of biomarkers is extensively discussed in the literature. Our group and others have shown that the amount of matrix metalloproteinase (MMP)-9 in the blood, a biomarker relevant for the prognosis of stroke [17] and cancer [18], can be critically affected by HMWH [19,20,21,22], an effect potentially impeding adequate treatment decisions. In contrast, other common anticoagulants such as EDTA, citrate, or even fractionated, i.e., low-molecular-weight heparin (LMWH), were less influencing [19,20]. Recently, we were able to show that the MMP-9-inducing effect of HMWH is not associated with a single cell type but requires the interaction of T cells and monocytes. Mechanistically, HMWH stimulates T cells to produce interleukin (IL)-16 and soluble intercellular adhesion molecule (sICAM)-1 which in turn activate the expression of MMP-9 by monocytes, an effect further enhanced by monocytic IL-8 in an autocrine feedback loop [20]. However, in these experiments, injection-grade heparin was applied exclusively. Though there is no fundamental difference between injection- and diagnostic-grade heparins (either HMWH or LMWH) in terms of structure, length, or composition, injection-grade heparins are applied as sodium or calcium heparin [23], while in blood collection tubes, lithium heparin is predominantly used [6]. Moreover, diagnostic-grade HMWH and LMWH are not subject to the strict purity/quality regulations that apply to heparins for human use [10,11]. Therefore, the potential differential effects of diagnostic-grade heparins remain to be assessed. In addition, the variety of known heparin contaminants and some less-frequently-used anticoagulants such as recombinant *Hirudo medicinalis*-derived Hirudin or Alteplase (a recombinant human tissue-type plasminogen activator (rt-PA) applied for intravenous thrombolysis due to its intervention in the final branch of the coagulation cascade) have not been considered yet. Thus, the aim of this study was to characterize the influence of these anticoagulants and a selection of known potential GAG-type heparin contaminants (HA, DS, CS, and OSCS) on monocytic MMP-9 expression and to assess the relevant molecular characteristics responsible for T cell activation.

## 2. Results

### 2.1. Supernatants of Anticoagulant-Treated Jurkat Cells Mediate Differential Effects on MMP-9 mRNA Expression in THP-1 Cells

In earlier experiments addressing the effect of anticoagulants on MMP-9 expression, HMWH and LMWH intended for systemic application were used [20]. Therefore, the effects of diagnostic-grade HMWH and LMWH (i.e., Enoxaparin) were assessed in an initial set of experiments.

#### 2.1.1. Diagnostic-Grade High- and Low-Molecular-Weight Heparin and Fondaparinux

**Figure 1:**
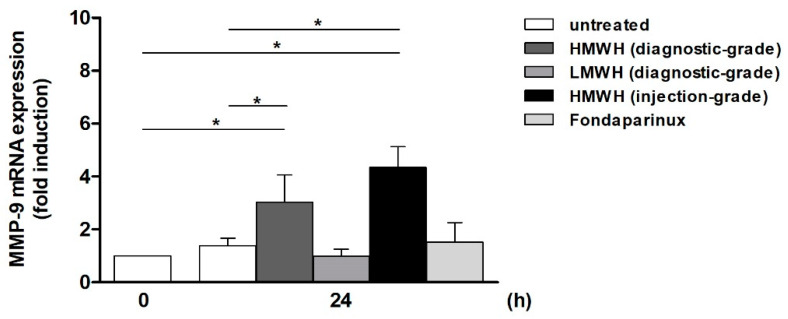
Supernatant of high-molecular-weight heparin (HMWH)-, but not low-molecular-weight heparin (LMWH)- or Fondaparinux-treated Jurkat cells, induces MMP-9 mRNA in THP-1 cells. Jurkat cells were starved for 24 h and then incubated for 24 h with injection- or diagnostic-grade HMWH (±50 IU per well; equates to 400 µg), diagnostic-grade LMWH (Enoxaparin; ±400 µg), or injection-grade Fondaparinux (±400 µg). Subsequently, the supernatant was harvested and transferred to starved THP-1 cells. Following a 24 h incubation phase, MMP-9 mRNA expression in the THP-1 cells was determined via quantitative (q)PCR. The MMP-9 expression value in starved THP-1 cells at 0 h (i.e., before the transfer of Jurkat supernatant) was set as 1 (n ≥ 4, mean ± SD; Mann–Whitney U-test, * *p* ≤ 0.05).

Following the stimulation of starved Jurkat T cells with diagnostic-grade HMWH and LMWH (i.e., Enoxaparin), as well as injection-grade HMWH as a positive control, the conditioned Jurkat supernatant was transferred to starved THP-1 cells (see Section 4.3.1 for experimental design). After 24 h, a significant HMWH-dependent induction of MMP-9 mRNA in comparison to the initial level at 0 h (diagnostic-grade: 3-fold, injection-grade: 4.3-fold) and the untreated 24 h control (diagnostic-grade: 2.2-fold, injection-grade: 3.2-fold) was observed. In the presence of diagnostic-grade Enoxaparin-treated medium, however, the MMP-9 expression remained virtually constant and was comparable to the expression level at 0 h (Figure 1).

For comparison, the synthetic anticoagulant Fondaparinux, a pentasaccharide mimicking heparin’s ATIII-binding site [24], was used in the same experimental setting. As equivalently reported for injection-grade LMWH [20], the presence of Fondaparinux for systemic purposes in the Jurkat medium did not result in a significant MMP-9 induction in THP-1 cells (Figure 1), indicating that both injection- and diagnostic-grade substances possess comparable features with regard to their T-cell-activating properties.

#### 2.1.2. Hirudin and Alteplase

**Figure 2:**
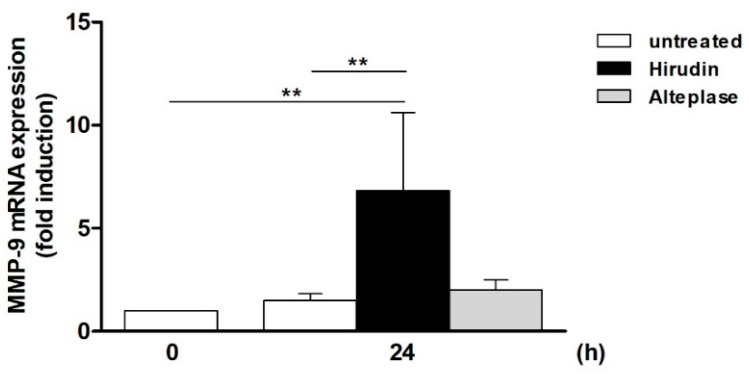
Supernatant of Hirudin-serum- and Alteplase-treated Jurkat cells induces MMP-9 mRNA in THP-1 cells. Jurkat cells were starved (24 h) and then incubated for 24 h with Hirudin serum (±220 µL per well; derived from a Hirudin blood collection tube) or Alteplase (±50,000 IU). Afterwards, the supernatant was transferred to starved THP-1 cells, and following a 24 h incubation phase, MMP-9 mRNA expression was determined (qPCR). The MMP-9 mRNA level in starved THP-1 cells at 0 h was set as 1 (n ≥ 5, mean ± SD; Mann–Whitney U-test, ** *p* ≤ 0.01).

To also evaluate the effects of clinically less common anticoagulants, we tested recombinant leech-derived Hirudin, a thrombin-binding and -inhibiting polypeptide, and the human rt-PA Alteplase that catalyzes the formation of plasmin, thus inducing fibrinolytic/thrombolytic processes. Using Hirudin, a significant increase in MMP-9 mRNA expression could be observed in relation to both the initial level at 0 h (approx. 7-fold) and the unstimulated 24 h control (4.5-fold; Figure 2). The application of Alteplase, however, did not result in a significant induction.

### 2.2. Supernatants of Heparin Contaminant-Treated Jurkat Cells Mediate Differential Effects on MMP-9 mRNA Expression in THP-1 Cells

The long carbohydrates HA, DS, and CS share some structural and chemical similarities with HMWH and have been described as contaminants in HMWH preparations [9,15], but do not possess anticoagulant properties. Thus, these GAGs were used for comparison in this study to assess the extent to which structure and charge influence MMP-9 expression (independent of intrinsic anticoagulant activity).

#### 2.2.1. Hyaluronic Acid, Dermatan Sulfate, and Chondroitin Sulfate

**Figure 3:**
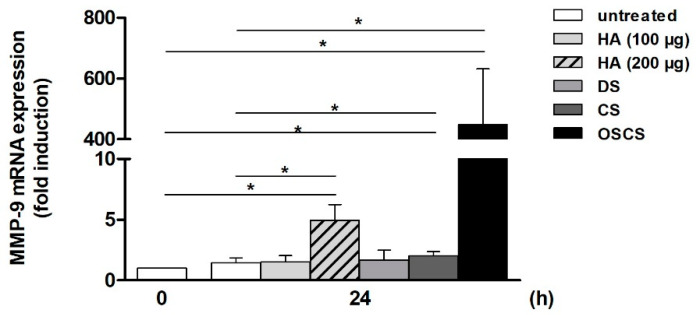
Effect of the supernatant of hyaluronic acid (HA)-, dermatan sulfate (DS)-, chondroitin sulfate (CS)-, and over-sulfated CS (OSCS)-treated Jurkat cells on MMP-9 mRNA in THP-1 cells. Jurkat cells were starved (24 h) and then incubated for 24 h ±100 or 200 µg HA, 400 µg DS, 400 µg CS, or 400 µg OSCS per well. Afterwards, the supernatant was transferred to starved THP-1 cells, and following a 24 h incubation phase, MMP-9 mRNA expression was determined (qPCR). The MMP-9 mRNA level in starved THP-1 cells at 0 h was set as 1 (n ≥ 3, mean ± SD; Mann–Whitney U-test, * *p* ≤ 0.05).

As shown in Figure 3, low dose HA-conditioned medium (i.e., treated with 100 µg) did not significantly alter MMP-9 levels, while higher amounts of HA (i.e., 200 µg) resulted in a significant elevation of MMP-9 in comparison to the unstimulated samples at 0 h and 24 h (approx. 5- and 3.5-fold increase, respectively). This indicates a dose-dependent effect of HA on T cells. Though DS and CS showed roughly comparable effects on MMP-9 mRNA expression (1.7- and 2-fold, respectively, when compared to the 0 h value), a significant increase could only be observed in the presence of CS.

#### 2.2.2. Over-Sulfated Chondroitin Sulfate

In the next step, the chemically modified CS derivative OSCS—that has been identified as an adulterant in HMWH preparations in 2007–2008 [2]—was analyzed. Interestingly, following the transfer of OSCS-treated Jurkat medium, THP-1 cells exhibited a dramatic, significant increase in MMP-9 mRNA in comparison to both the controls (0 h: approx. 450-fold, 24 h: approx. 320-fold; Figure 3).

### 2.3. Characterization of Cytokine/Chemokine Patterns Expressed in Response to Anticoagulants and Contaminants

Following the stimulation with injection-grade HMWH for 24 h, starved T cells are characterized by the expression of specific MMP-9-inducing cytokine patterns [20]. To test which T-cell-derived mediators (e.g., cytokines/chemokines, growth factors, and others) were involved in the elevation of monocytic MMP-9 mRNA in response to the anticoagulants and contaminants tested in the present study, the respective T cell supernatants were analyzed with the Proteome Profiler Human (XL) Cytokine Array.

#### 2.3.1. Anticoagulants: HMWH, LMWH, Hirudin, and Alteplase

In Table 1, the mediators present in the supernatant of anticoagulant-stimulated T cells are given. Several factors, such as macrophage migration inhibitory factor (MIF), Serpin E1 (also known as plasminogen activator inhibitor 1), or various binding proteins (BP, e.g., IL-18-BP, insulin-like growth factor-BP-2 and -3, or retinol-BP-4), were observed in various approaches, including those without detectable MMP-9 induction (i.e., in the presence of injection- or diagnostic-grade LMWH, Fondaparinux, or in the untreated controls). This indicates that these factors do not possess MMP-9-inducing capacity under the applied conditions and were thus not considered further. For comparison, monocytes were incubated with the supernatant of injection-grade HMWH-treated T cells. As expected from our previous study [20], this condition induced the secretion of sICAM-1 and IL-16. In response to diagnostic-grade HMWH, however, the specific secretion of sICAM-1 and IL-22 could be detected, suggesting that the particular composition of different HMWH batches may have an influence on the cytokines specifically secreted by T cells. Though Hirudin-conditioned T-cell supernatants were characterized by the presence of a variety of different mediators, including sICAM-1 and IL-16, no specific cytokine/chemokine profile could be identified due to a high inter-experimental variation of the detected factors. Together, our data indicate that more than one combination of mediators may be involved in the induction of MMP-9 in monocytic cells—with sICAM-1 as a central player in these varying combinations.

#### 2.3.2. Contaminants: HA, DS, CS, and OSCS

Equivalently, the factors secreted in response to heparin contaminants were identified (Table 2). Unexpectedly, no differential cytokine expression profiles could be detected among the supernatants of untreated controls, 100 µg HA-, and 200 µg HA-stimulated T cells. This argues for an involvement of less usual (or other types of) soluble mediators that cannot be assessed with the array applied.

However, both DS and CS led to the release of sICAM-1, though only CS-conditioned supernatant had a significant effect on monocytic MMP-9 mRNA. Considering that both GAGs induced a roughly comparable increase in MMP-9 in terms of absolute numbers (see Section 2.2.1), this phenomenon suggests that CS and DS may generally exert a similar impact on T cells with only subtle differences.

OSCS, the contaminant/adulterant responsible for the dramatic induction of MMP-9, provoked the secretion of the most substantial and variable combination of T-cell-derived factors, i.e., sICAM-1, IL-5, IFN-γ, and kallikrein 3. These data further support the assumption that monocytic MMP-9 production may be induced by a variety of different mediators, with sICAM-1 being a crucial component.

### 2.4. Induction of MMP-9 mRNA Expression in Monocytic THP-1 Cells by T-Cell-Derived Mediators

**Figure 4:**
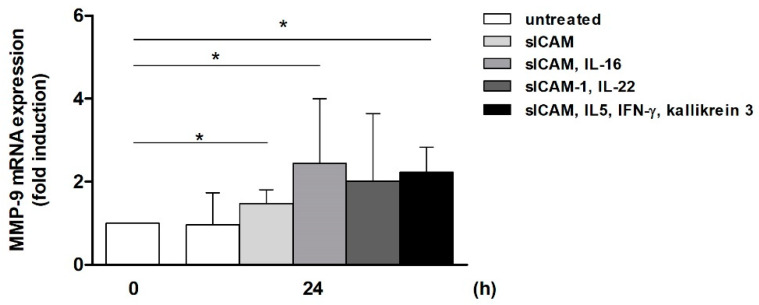
Induction of MMP-9 expression by T-cell-derived mediators. A total of 0.5 × 10^6^ THP-1 cells/well were starved for 24 h. Subsequently, cells were stimulated for 24 h with combinations of soluble intercellular adhesion molecule (sICAM)-1, interleukin (IL)-16, IL-22, IL-5 (5 ng/mL each), interferon (IFN)-γ (10 ng/mL), and kallikrein 3 (1 µM). MMP-9 mRNA expression was determined using qPCR. The MMP-9 mRNA level in starved THP-1 cells at 0 h was set as 1 (n = 3, mean ± SD; Mann–Whitney U-test, * *p* ˂ 0.05).

To confirm the effect of the T-cell-derived mediators on monocytic MMP-9 expression, THP-1 cells were stimulated with (combinations of) sICAM-1 and the identified cytokines. As reported before [20], stimulation with sICAM-1 has a significant, but rather small, absolute effect on MMP-9 (approx. 1.5-fold induction, Figure 4). In combination with either IL-16 or IL-5, IFN-γ, and kallikrein 3, an enhanced significant stimulatory effect on MMP-9 mRNA could be demonstrated (2.4-fold and 2.2-fold, respectively). IL-22, though not statistically significant, showed a comparable tendency (2-fold induction; Figure 4). Together, these results support the assumed synergistic influence of the identified messenger molecules on MMP-9. However, in the case of the OSCS-induced mediators, which provoke only a mild induction that is not comparable to the effect induced by the OSCS-conditioned medium, further mechanisms have to play a role and remain to be elucidated.

## 3. Discussion

The influence of different anticoagulants on blood cells is a crucial aspect of medical applications and extensively discussed in the literature, since such effects may involve various clinical complications and compromise the diagnostic validity of blood analyses. MMP-9, also known as gelatinase B, is a member of the zinc-dependent matrix-metalloproteinase family [25]. As part of a complex regulatory network [26], MMP-9 predominantly degrades extracellular matrix (ECM) components (e.g., collagen IV, V, VII, X, and XIV, gelatin, aggrecan, fibronectin, laminin, and others) [27]. By the proteolytic cleavage of precursor proteins, it is also involved in the activation of cytokines (e.g., TNF, TGF-β), clotting-associated factors (e.g., fibrinogen, plasminogen), and proteases (including MMP-1 and -2, and, via autoregulation, MMP-9) [28]. In blood, MMP-9 is mainly produced by monocytes [29], and its expression is a relevant biomarker, e.g., for the prognosis of stroke [17]. Currently, due to its role in angiogenesis, invasiveness, metastasis, and ECM reorganization, the suitability of MMP-9 as a biomarker is also extensively investigated in different types of cancers [18], cardiovascular diseases [30], and pregnancy complications such as preeclampsia [31]. It has been shown before that MMP-9 determination may be distorted depending on the anticoagulant used during blood withdrawal. While EDTA, citrate, or injection-grade LMWH has a rather small and thus neglectable influence [19,20], the unfractionated HMWH significantly enhances MMP-9 values over time [19,20,21,22], an effect potentially impeding adequate treatment decisions. Mechanistically, HMWH appears to activate T cells, resulting in the secretion of IL-16 and sICAM-1, which in turn induce MMP-9 expression in monocytes. This effect is further supported by monocytic IL-8 in an autocrine feedback loop [20]. However, several relevant aspects have not been adequately considered yet, e.g., the impact of (i) less frequently applied anticoagulants (Hirudin or Alteplase), (ii) the variety of described HMWH contaminants and adulterants (HA, DS, CS, and OSCS), and (iii) some experimental details (e.g., the application of injection-grade vs. diagnostic-grade heparin). Thus, this study was conducted to address these aspects and to further characterize the influence of anticoagulants and related parameters on T cell cytokine production and subsequent monocytic MMP-9 expression.

### 3.1. Specific Anticoagulants Can Act as Inducers of MMP-9 mRNA Expression

In the initial phase of the current study, the anticoagulants HMWH and LMWH of diagnostic quality, as well as Fondaparinux, Hirudin, and Alteplase were tested. Heparins are naturally occurring GAGs of variable lengths and MW (up to 40 kDa). They consist of variably sulfated disaccharide units, i.e., glucosamine, glucuronic acid, or iduronic acid [32] that efficiently inhibit both FXa and FIIa via ATIII [3]. Despite their chemical similarity, fractionated heparin possesses lower charge and reduced intensity of unspecific binding to cellular proteins [33] due to its significantly reduced MW (approx. 5 kDa in average [1]) and chain length [32]. In addition to the application of both unfractionated and fractionated sodium heparin for therapeutic purposes [7], unfractionated heparin is widely used in lithium heparin blood collection tubes for blood withdrawal [34] and the subsequent diagnostic determination of various biochemical parameters [35] and biomarkers [36]. With respect to the structure of the glycosaminoglycans, however, there is no intrinsic difference among the injection- and diagnostic-grade heparins. The level and composition of impurities, though, may differ since diagnostic-grade heparins are subject to less strict quality regulations. Fondaparinux is a synthetically manufactured small molecule (approx. 1.8 kDa) that corresponds to the antithrombin-binding pentasaccharide unit of heparin, selectively associates with ATIII, and thus specifically inhibits FXa. Since it is not of animal origin, Fondaparinux has no significant batch-to-batch variations or risk of transmissible diseases due to contamination with animal pathogens [37]. Moreover, in contrast to heparins, Fondaparinux has no direct effect on the thrombin activity or the release of tissue factor pathway inhibitor [38]. Its clinical application also has some advantages over LMWH, e.g., a further reduced degree of interactions with proteins, an improved prevention of deep vein thrombosis [38], fewer allergic reactions [39], and a reduced number of bleeding events [40]. Hirudin, actually a recombinant version of the natural *Hirudo medicinalis* salivary gland-derived anticoagulant, is an acidic 7 kDa single-chain polypeptide consisting of 65 amino acids. Following its association with the B chain of thrombin, Hirudin blocks both the active center and (by inducing conformational changes) the specificity region of thrombin, thus acting as a bi-functional inhibitor [41]. In comparison to the other anticoagulants, Hirudin is applied less often due to some disadvantages, e.g., high costs and an increased tendency to cause bleeding [42]. However, it is still indicated in the clinic for prophylaxis or the therapy of thrombotic diseases [41,42], and Hirudin tubes are electively used for specific hematological and serological purposes [43,44]. Alteplase, a recombinant version of human t-PA, is a glycosylated single-chain serine protease of approx. 70 kDa that comprises 527 aa [45]. Alteplase is especially applied for intravenous thrombolysis (e.g., in acute ischemic stroke [46] or pulmonary embolism [47]) due to the efficient conversion of plasminogen to plasmin in the presence of fibrin clots [45,48].

Our results revealed that HMWH- and Hirudin-conditioned T cell medium possess statistically significant MMP-9 mRNA-inducing capacity when transferred to monocytes, while LMWH-, Fondaparinux-, and Alteplase-conditioned T cell medium did not. As expected, elevation of MMP-9 mRNA was well reflected on the protein level as observed in initial control experiments using HMWH. These results are in good agreement with the results of our previous studies, in which injection-grade HMWH, but not LMWH, EDTA, or citrate, mediated an increase in both MMP-9 mRNA and protein expressions [19,20]. However, in absolute numbers, injection-grade HMWH and Hirudin provoked stronger effects than diagnostic-grade HMWH (4–10-fold [20] and 7-fold vs. 3-fold induction, respectively), indicating that the biological relevance may strongly vary among the respective substances. Moreover, the existence of anticoagulant activity as such is not a sufficient prerequisite for the MMP-9-inducing capacity of a given substance, as evidenced by the negative results of EDTA, citrate [20], LMWH, Fondaparinux, and Alteplase.

### 3.2. Heparin Contaminants Can Intensify Anticoagulant-Induced MMP-9 Expression

In the next step, known heparin contaminants and adulterants, including HA [9], DS, CS, and OSCS [49], were analyzed. Like heparin, HA is a long, unbranched, and polar GAG (>1000 kDa in its HMW form). It is predominately produced by mesenchymal cells, composed of numerous repeated sulfate-free disaccharides, i.e., D-glucuronic acid (GlcA) and N-acetyl-D-glucosamine (GlcNAc), and has been reported to possess antiangiogenic and immunosuppressive capacity [50]. As a major component of the ECM, HMW-HA modulates key features such as cellular signaling, adhesion, proliferation/differentiation, wound healing/tissue regeneration, and inflammation [50,51]. CS, in turn, generally consists of various numbers of GlcA and N-acetyl-galactosamine (GalNAc) disaccharides (MW: up to 40 kDa; approx. 20 kDa in average [52]) with one or two sulfate groups at the GalNAc (C4 and/or C6 position) [53]. DS, also known as CS-B and closely related to CS, consists of L-iduronic acid (IdoA, derived from GlcA by isomerization) and GalNAc [54]. In DS (mean MW: 20 kDa [55]), sulfation typically occurs at the C2 position of IdoA and the C4 position of GalNAc [56]. Both CS and DS are components of the polysaccharide chains covalently attached to the membrane-bound core proteins. They are usually found in hybrid structures within the same chain and—as HA—are involved in the regulation of cellular functions involving the interaction with the ECM, including proliferation and differentiation, adhesion, morphogenesis, and immunity [54,57]. DS and CS are present in large amounts in animal tissues, including the porcine intestinal mucosa, which is the main source of heparin. Due to the structural and physicochemical similarity between heparin and DS/CS, both are co-isolated with HMWH and have to be regarded as naturally occurring, process-related impurities [58]. CS oversulfation, however, results from the artificial chemical addition of extra sulfate residues at the C2 and C3 positions of the GlcA and at the remaining C4 or C6 positions of the GalNAc [53]. The resulting (patho-)physiological effects appear to be largely independent of the original CS source [59]. As in the case of other suitable GAGs, the anticoagulant activity of OSCS relies on the amount of negative charge inhering in the degree of sulfation. Mechanistically, these highly charged molecules activate the contact system [60]. In contrast, lowly charged GAGs, such as naturally occurring chondroitin 4- [61] or 6-sulfate [53], do not possess perceptible anticoagulant capacity. However, it has been proposed that beyond sulfation, other subtle structural interactions may also play a role [62]. In comparison to heparin, OSCS inhibits the coagulation cascade only moderately by potentiating heparin cofactor II activity, thus inhibiting FIIa [61,62]. By activating plasminogen, OSCS also supports the fibrinolytic processes [63]. In combination with heparin, however, OSCS appears to provoke super-additive effects on both anticoagulation and bleeding, as reported for contaminated HMWH in animal models of thrombosis and bleeding, respectively [64].

Our experiments revealed that lower amounts of HA (i.e., 100 µg) as well as DS have no significant impact on T cell activation and the subsequent MMP-9 mRNA expression. Despite statistical significance, CS stimulation resulted in only a weak effect (2-fold induction) that was roughly comparable to DS (1.7-fold), which suggests a limited biological impact on MMP-9. Larger amounts of HA (200 µg), however, led to a stronger (5-fold) significant MMP-9 elevation, indicating a dose-dependent effect. Here, the molecular characteristics responsible for the secretion of T-cell-derived MMP-9-inducing mediators appear to be effective only in higher concentrations, but insufficient in lower amounts. The most impressive effect on MMP-9 mRNA levels (and protein levels, as demonstrated in single control experiments) was observed when the highly sulfated OSCS was used as a stimulus (450-fold induction). Thus, it is reasonable to assume that the length and—presumably even more important—charge of the respective GAGs might play a role. In the case of long-chained HA, low amounts could be ineffective due to the absence of sulfate groups, an effect that may be balanced in higher concentrations by its intrinsic polarity [50]. The moderate sulfation [65] and the reduced MW (when compared to HA) of both DS and CS may account for their detectable, in part even significant, but nevertheless limited effectiveness. Thus, the similarly long HMWH may act as a more potent stimulus due to the high degree of sulfation [1]. In addition, the specific length of the disaccharide chains also appears to be crucial for T-cell-activating capacity, since the significantly shorter LMWH had no effect (despite the comparable degree of sulfation per disaccharide). OSCS, in turn, may act as an unmatched inducer due to its length and the artificially enhanced degree of sulfation.

Collectively, our data suggest that the length and charge/polarity of substances with T-cell-activating capacity (like HMWH and OSCS) may represent the relevant molecular features leading to the secretion of mediators activating MMP-9 expression, while smaller and lesser charged substances (e.g., Fondaparinux, LMWH) have no such effect. At the molecular level, a mechanism involving the activation of specific proteins/receptors on the surface of T cells by long highly charged GAGs is conceivable (e.g., by crosslinking). In the case of the acidic polypeptide Hirudin, which induced quite variable cytokine profiles among different experiments, the relatively low molecular weight appears to be of minor importance, though related aspects such as conformation may play a role, while HA—for which no specific cytokine profile could be observed—appears to utilize a completely different set of mediators. These issues need to be clarified in further studies.

Nonetheless, in the current batches of heparin, contamination with (residual) DS, CS, or HA, potentially retained in the course of the isolation and purification process [58], appears to be neglectable regarding biomarker validity due to the limitation of natural contaminants to the low, officially tolerated degree. Moreover, the supplementation of heparin with semi-synthetically produced adulterants, such as OSCS, is prohibited. This is ensured by strict quality controls [10,11] and reflected by the effectively reduced amounts of GAG impurities due to continuously improved purification processes [49]. Thus, the MMP-9-inducing capacity of current HMWH may predominantly depend on its intrinsic molecular features (though potential additive effects cannot be excluded), and the strength of the outcome may vary among different preparations of heparin because of their composition and purity. Accordingly, when using injection-grade HMWH as a positive control in the present study, we observed lower effect sizes than in our previous work (4.3-fold vs. 10-fold [20]), where we used heparin batches that were acquired in a time when contaminants and adulterants, such as OSCS, were more prevalent.

Systemically applied, even pure heparin possesses potential adverse effects. Heparin-induced thrombocytopenia (HIT) type II, i.e., the depletion of platelets via the antibody recognition of large thrombocyte–heparin complexes resulting in thrombus formation, is the most prominent heparin-associated risk involving varying clinical complications with different severity. Such effects, however, were well characterized over the last decades and can be effectively treated [5]. Interestingly, Arteparon—a polysulfated CS used for the treatment of degenerative joint disease—also caused an HIT-like disease and other adverse effects in several cases and was removed from the German market in the early 1990s [66,67], indicating that the adverse effects of highly sulfated GAGs are a recurrent problem.

### 3.3. Soluble Mediators Link T Cell Activation with Monocytic MMP-9 Expression

As stated above, T-cell-derived soluble mediators are the link between the stimulating agent and monocytic MMP-9 expression in our experimental setting. Several mediators, such as MIF, Serpin E1, or various binding proteins, however, did not contribute to monocytic MMP-9 expression since they were also detected in the approaches without a detectable MMP-9 induction. This is in good agreement with our previous experiments, in which the direct stimulation of monocytes with MIF and Serpin E1 did not result in MMP-9 induction [20]. In contrast, sICAM-1 was a part of the distinct cytokine profiles in all experiments exhibiting (significantly) induced MMP-9 expression levels. Thus, it appears to be ubiquitously released by activated T cells and a key factor in the MMP-9-inducing mediator cocktails, at least under the conditions tested. In other settings, e.g., in rIFNβ-1b-treated patients with relapsing-remitting multiple sclerosis, a negative correlation between sICAM-1 and MMP-9 has been described [68]. However, sICAM-1 alone—as observed in the DS- and CS-containing approaches—turned out to be less effective as demonstrated by low absolute (and in the case of DS, not even significant) MMP-9 induction values (approx. 1.5-fold), emphasizing the necessity of additional factors. In our previous study, sICAM-1 proved to be the only mediator that is able to induce MMP-9 alone, but only to a similarly limited extent [20].

Our results also demonstrate a difference between the cytokines induced following the stimulation of T cells with unfractionated injection-grade sodium heparin (IL-16) and diagnostic-grade lithium heparin (IL-22). Future studies should address whether this variation depends on the cations used, the differences in the quality of heparins applied for different purposes, or the variations among heparin batches. The combination of IL-16 or IL-22 with sICAM-1 provoked a roughly comparable MMP-9 induction (2.4- vs. 2-fold, respectively), though statistical significance was not reached in the case of IL-22. While IL-22-dependent production of MMP-9 has been observed during the migration and invasion of gastric [69] and breast cancer cells [70], the limited effect observed in THP-1 cells may be ascribed to the utmost weak expression of one of the two receptor subunits (IL-22R1) necessary for IL-22 signaling in THP-1 [71]. Nonetheless, our data suggest that various combinations of cytokines have a distinct impact on monocytic gene expression, irrespective of the substance that provoked the secretion of these factors. Of course, other influencing parameters, such as the dose and proportion of the mediators as well as the temporal secretion profile, may play a role within this context.

OSCS yielded the most drastic enhancement of MMP-9 expression, based on the induction of sICAM-1, IL-5, IFN-γ, and kallikrein 3. Again, sICAM-1 cooperates with another interleukin, substantiating the diversity of interleukins that may contribute to the regulation of MMP-9 [72]. IFN-γ and kallikrein 3 may support the induction of MMP-9, though definitely not to the extent observed in the presence of OSCS-conditioned medium, suggesting that additional mediators or mechanisms have to play a major role in mediating the OSCS-dependent effect. While IFN-γ has already been described as a potent MMP-9-inducing cytokine [72], the detection of kallikrein 3 (also known as prostate-specific antigen, PSA) is somewhat surprising since it is regarded as a prostate-specific marker [73] that—to our knowledge—has not been described in the regulation of MMPs yet. Interestingly, kallikrein 3 also possesses the potential to act on the coagulation cascade by activating plasminogen, and this effect is potentiated by GAGs such as heparin and DS [74].

### 3.4. Summary

**Figure 5:**
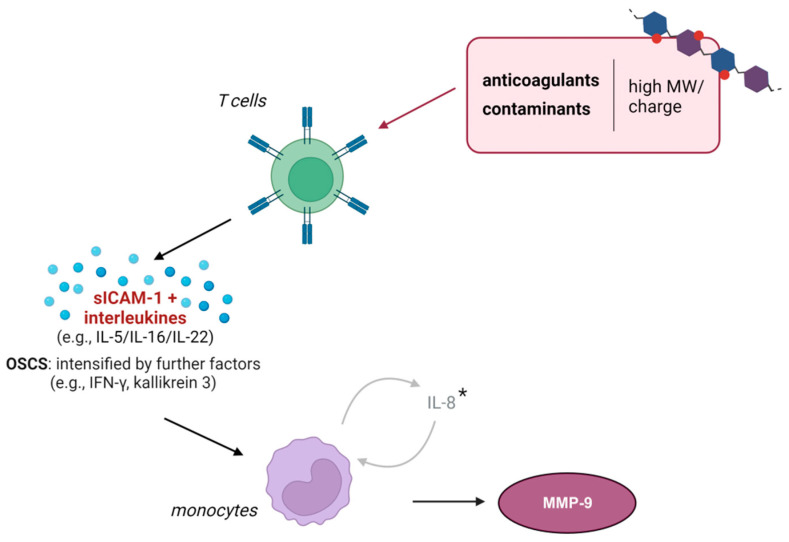
Extended model of monocytic MMP-9 expression during anticoagulation with specific agents. Our results suggest that in the presence of anticoagulants and heparin contaminants with high charge and molecular weight (MW), T cells secrete sICAM-1 in combination with different interleukins (e.g., IL-5, -16, or -22). In the presence of OSCS, T cells secrete additional mediators (IFN-γ, kallikrein 3) that intensify monocytic activation. * Injection-grade HMWH-induced sICAM-1 and IL-16 activate an additional IL-8-dependent positive autocrine feedback loop [20]. Created with BioRender.com.

Taken together, when challenged with suitable, i.e., large and highly polar/charged (esp. GAG-type) anticoagulants or heparin contaminants, T cells secrete specific activating cytokine and chemokine cocktails, which induce the expression of MMP-9 in monocytic cells (Figure 5). This mechanism may confound the measurement of MMP-9 and thus its validity as a biomarker.

Equivalent mechanisms may distort the expression of other relevant biomarkers. Hence, for diagnostic purposes, the selected anticoagulants should have a lower MW as well as charge and should have been tested for a potential influence on the respective biomarker(s) of interest. This consideration should also be taken into account during systemic application to avoid undesired adverse effects of anticoagulation.

## 4. Materials and Methods

### 4.1. Cell Lines and Cell Culture Conditions

Jurkat (human T cell leukemia cell line, ACC-282) and THP-1 cells (human acute monocytic leukemia cell line, ACC-16; both DSMZ, Braunschweig, Germany) were maintained in Roswell Park Memorial Institute 1640 medium supplemented with 100 U/mL penicillin, 100 mg/mL streptomycin (Biochrom, Berlin, Germany), and 10% FCS (Sigma Aldrich, St. Louis, MO, USA) at 37 °C in a humidified atmosphere containing 5% CO_2_. Cell culture experiments were performed in 6-well plates (Sarstedt, Nümbrecht, Germany) with 2 mL medium/well. For supernatant transfer experiments, Jurkat and THP-1 cells were starved (0.5–1% FCS) for 24 h in the absence of antibiotics. Starved cells without further stimulation were used as negative controls.

### 4.2. Reagents

Diagnostic-grade HMWH and LMWH (i.e., Enoxaparin) as well as Hirudin-containing blood collection tubes (cat. # 04.1959.001) were obtained from Sarstedt (Nümbrecht, Germany). As a control, injection-grade HMWH (Rotexmedica, Trittau, Germany; pharmaceutical central number (PCN): 3862340) was used. The synthetic anticoagulant Fondaparinux was purchased from Aspen (Durban, South Africa; PCN: 2450612), the rt-PA Alteplase from Boehringer Ingelheim (Ingelheim, Germany; PCN: 03300636), DS and CS from Sigma Aldrich (St. Louis, MO, USA; cat. # C3788 and C9819), OSCS from Serva Electrophoresis (Heidelberg, Germany; cat. # 31254), and HA from Bausch + Lomb (Rochester, NY, USA; PCN: 07707033). With the exception of Hirudin (which was applied as Hirudin serum), all substances were dissolved, and dilutions were prepared using *aqua ad iniectabilia* (B. Braun, Melsungen, Germany; PCN: 03710653).

### 4.3. Cell Culture Experiments

#### 4.3.1. Supernatant Transfer Experiments

**Figure 6:**
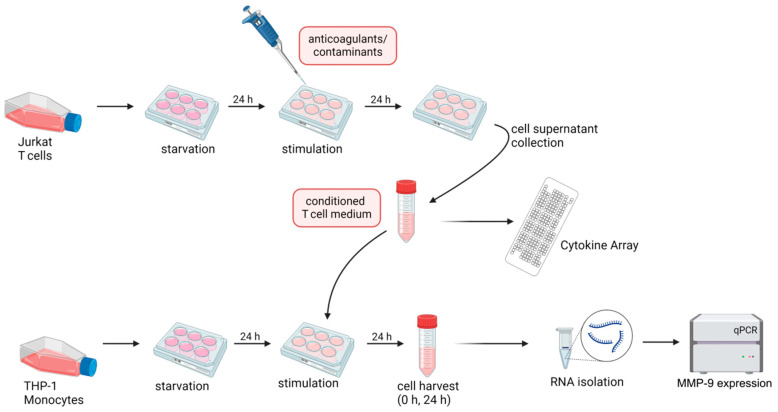
Experimental Design. Jurkat T cells were cultivated in starvation medium for 24 h and subsequently stimulated with anticoagulants or contaminants. After 24 h, the conditioned Jurkat medium was transferred to starved THP-1 monocytic cells. In parallel, cytokines secreted in response to the respective stimuli were detected in aliquots of the Jurkat supernatant using the Proteome Profiler Human (XL) Cytokine Array. The MMP-9 mRNA expression in THP-1 cells was assessed via qPCR following a 24 h incubation phase in the T-cell-derived medium. Created with BioRender.com.

For cell supernatant transfer experiments, 2 × 10^6^ Jurkat T cells/well were starved for 24 h (see Section 4.1) and then stimulated for 24 h with 50 international units (IU; i.e., 400 µg [75,76]) HMWH, 400 µg Enoxaparin, Fondaparinux, DS, CS, OSCS (to apply amounts comparable to HMWH), 220 µL Hirudin serum (volume as applied in our previous study [20]), 100 or 200 µg HA (to compensate for its higher molecular weight), or 50,000 IU Alteplase (to reflect the higher specific activity that is applied therapeutically). Afterwards, the Jurkat supernatant was collected, 2 × 10^6^ starved monocytic THP-1 cells/well were stimulated for 24 h with the conditioned medium, and the MMP-9 expression was determined (Figure 6). In parallel, the presence of cytokines and chemokines secreted in response to the respective anticoagulant/contaminant was analyzed in an aliquot of the supernatant using the Proteome Profiler Human XL Cytokine Array (R&D Systems, Minneapolis, MN, USA; see Section 4.5).

#### 4.3.2. Cytokine Stimulation Experiments

Following starvation for 24 h, 0.5 × 10^6^ THP-1 cells/well were stimulated for 24 h with human recombinant sICAM-1, IL-5, IL-16, IL-22 (5 ng/mL each), IFN-γ (10 ng/mL; Peprotech, Hamburg, Germany), or kallikrein 3 (1 µM; R&D Systems), either alone or in combination. Following stimulation, cells were collected, and MMP-9 mRNA expression levels were assessed [20].

### 4.4. RNA Extraction, cDNA Synthesis, and Quantitative (q)PCR

Cell lysis and RNA isolation were performed using the RNeasy Mini Kit (Qiagen, Hilden, Germany) [77]. RNA was eluted in 60–80 μL RNase-free water, and concentration as well as quality were assessed using the NanoDrop ND-1000 photometer at 260/280 nm (Peqlab, Erlangen, Germany). For complementary DNA (cDNA) synthesis, 1 μg total RNA per sample was reverse transcribed (Prime Script RT Master Mix; Takara, Saint-Germain-en-Laye, France) as summarized in [78]. Subsequently, cDNA samples were stored at −80 °C. Quantitative polymerase chain reaction (qPCR) was performed using the LightCycler 480 II (Roche Diagnostics, Mannheim, Germany) [19]. In short, the MMP-9 analysis was based on the QuantiTect Custom Assay (Qiagen; forward primer: 5′-TCCAGTACCGAGAGAAAG-3′, reverse primer: 5′-CAGGATGTCATAGGTCACGTAG-3′, and hybridization probe: 5′-non-fluorescent quencher-GGAGTGAGTTGAACCAG-6-carboxyfluoroscein-3′). Normalization was performed using sequence-specific fluorescent resonance energy transfer probes for the housekeeping gene glyceraldehyde-3-phosphate dehydrogenase (GAPDH; forward primer: 5′-TGCTGAGTATGTCGTGGAGTC-3′, reverse primer: 5′-GGATGCAGGGATGATGTTCT-3′; donor hybridization probe: 5′-GACAACTTTGGTATCGTGGAAGGACTCATGACCACA-fluorescein thiocyanate-3′, and acceptor hybridization probe: 5′-Cy5.5-CTGAGCGTGGCTATTCCTTCGTGACTACTG-phosphate-3′). For the determination of GAPDH, 50 ng cDNA was mixed with 4 pmol hybridization probes, 10 pmol of each primer, and 10 µL LightCycler 480 Probes Master (Roche Diagnostics). H_2_O was added to a final volume of 20 μL. PCR conditions were as follows: initial hot start incubation (95 °C, 5 min), denaturation (95 °C, 10 s), annealing (56 °C, 30 s for MMP-9; 59 °C, 30 s for GAPDH), and extension (72 °C, 30 s).

For the calculation of cDNA concentration, plasmid standards were generated and applied in serial dilutions (1 mg/μL to 100 fg/μL) as described in [79].

### 4.5. Proteome Profiler Array

The Proteome Profiler Human Cytokine Array Kit or the Proteome Profiler Human XL Cytokine Array (R&D Systems) were utilized as described in [80] to identify cytokines and chemokines secreted by starved Jurkat cells in response to anticoagulants or known contaminants. In short, array membranes (consisting of capture antibodies spotted in duplicate on nitrocellulose) were incubated with 1.5 mL supernatant (derived from stimulated Jurkat cells) and a cocktail of biotin-coupled capture antibodies (15 µL; 24 h, 4 °C). Subsequently, membranes were washed and incubated for 30 min with 1.5 mL Streptavidin–HRP solution. Membrane-bound cytokines and chemokines were detected using the chemiluminescent detection reagent provided and the bio-imaging system ECL Chemostar (Intas Science Imaging, Göttingen, Germany).

### 4.6. Statistical Analysis

Data were described as means ± standard deviation (SD). The Mann–Whitney U-test was applied to analyze differences among mRNA levels following stimulation using GraphPad Prism version 5.02 (GraphPad Software, La Jolla, CA, USA).

## 5. Conclusions

Our results indicate that in response to a variety of (predominantly GAG-type) anticoagulants and contaminants with a high MW and, above all, a high charge density (but independent of genuine anticoagulant activity), T cells react with the expression of specific activating cytokine/chemokine cocktails, which induce the expression of MMP-9 (and presumably other relevant biomarkers) in monocytic cells. This effect potentially impairs the diagnostic validity of MMP-9 as a biomarker and argues for the selection of anticoagulants with reduced MW and charge, wherever possible.

## Figures and Tables

**Table 1:** Cytokine/chemokine profiles expressed by Jurkat T cells following stimulation with high-molecular-weight heparin (HMWH) or low-molecular-weight heparin (LMWH), Fondaparinux, and Hirudin as determined by the Proteome Profiler Human (XL) Cytokine Array (Fondaparinux and Alteplase, n = 2; all other experiments, n = 3).

Anticoagulant	Secreted Mediators
HMWH (injection-grade)	sICAM-1, IL-16
LMWH (injection-grade)	-
HMWH (diagnostic-grade)	sICAM-1, IL-22
LMWH (diagnostic-grade)	-
Fondaparinux	-
Hirudin	diverse factors ^1^
Alteplase	-

^1^ In Hirudin-conditioned medium, the cytokine profile showed a high variability among different experiments. Detected mediators include soluble intercellular adhesion molecule (sICAM)-1, interleukin (IL-)16, B cell activating factor, chitinase-3-like protein 1, cystatin C, and chemokine (C-C motif) ligand 5.

**Table 2:** Cytokine/chemokine profiles expressed by Jurkat T cells following stimulation with the HMWH-contaminating glycosaminoglycans hyaluronic acid (HA), dermatan sulfate (DS), chondroitin sulfate (CS), and over-sulfated CS (OSCS) as determined by the Proteome Profiler Human XL Cytokine Array (n = 3).

Contaminant	Secreted Mediators
HA (100 µg)	-
HA (200 µg)	-
DS	sICAM-1
CS	sICAM-1
OSCS	sICAM-1, IL-5, IFN-γ, kallikrein 3

## Data Availability

The original contributions presented in this study are included in the article; further inquiries can be directed to the corresponding author.
